# A reassessment of the vitamin D requirements of modern broiler genotypes

**DOI:** 10.3382/ps/pey350

**Published:** 2018-08-24

**Authors:** P Sakkas, S Smith, T R Hill, I Kyriazakis

**Affiliations:** 1 Agriculture, School of Natural and Environmental Sciences, Newcastle University, Newcastle on Tyne, NE1 7RU, UK; 2 Institute of Cellular Medicine, Faculty of Medical Science, Newcastle University, Newcastle on Tyne, NE1 7RU, UK

**Keywords:** vitamin D, 25-OH-D3, broiler, growth rate, bone mineralization

## Abstract

We hypothesized that performance and bone mineralization of 2 broiler lines will benefit from increasing vitamin D (**vitD**) supplementation above current commercial levels and by partial substitution of D_3_ by 25-OH-D_3_. Male Ross 308 and 708 chicks (n = 576), were offered diets with low (**LD**; 1,000), medium (**MD**; 4,000) or high levels of D_3_ (**HD**; 7,000 IU/kg), and medium levels of vitD where the majority of D_3_ was substituted by 25-OH-D_3_ (**25MD**; 1,000 D_3_+3,000 25-OH-D_3_ IU/kg). Performance was measured at the end of starter (day 10), grower (day 24), and finisher periods (day 38). Three birds per pen were dissected at the end of each period to assess tibia and femur ash percentage (%), ash weight, bone breaking strength (**BBS**), and serum levels of 25-OH-D_3_. Remaining birds were gait scored (**GS**) at day 37 of age. Genotype and diet did not interact for any trait, whilst performance was not affected by diet. Ross 708 had lower body weight (*P <* 0.005), higher feed conversion ratio over the grower period (*P <* 0.05), similar levels of 25-OH-D_3_, but higher GS (*P <* 0.05) than Ross 308. Serum 25-OH-D_3_ levels were affected by diet at the end of the starter and grower periods (*P <* 0.05), being lowest for LD and highest for 25MD. Diet affected GS (*P <* 0.01), being higher in LD than 25MD. Femur ash % was higher at the end of the starter and grower periods for 25MD than LD and for both HD and 25MD than LD (*P <* 0.05). Femur and tibia ash weight were higher for 25MD in comparison to LD birds (*P <* 0.05) at the end of the grower period. Femur and tibia BBS were higher (*P <* 0.05) for 25MD in comparison to LD at the end of the grower and finisher periods, respectively. Overall, effects of vitD supply were more pronounced for femur than for tibia mineralization. Results do not suggest supplementation of vitD above current maximum levels and support partial substitution by 25-OH-D_3_.

## INTRODUCTION

Genetic selection of broiler chickens has amongst other characteristics improved substantially growth rate and feed conversion ratio (**FCR**) (Siegel, 2014), and has shifted focus towards breast muscle rather than leg muscle yield, moving the center of gravity of the bird forward (Corr et al., 2003a). Higher growth rates have been suggested as a cause for the increasing occurrence of skeletal abnormalities of the locomotory system observed in broilers (Kestin et al., 2001; Williams et al., 2004), although genetic selection for improving leg health may reduce their incidence, despite somewhat unfavorable genetic correlations with growth rates (Kapell et al., 2012).

Vitamin D (**vitD**) is involved in skeletal integrity through the stimulation of the expression of genes in the small intestine which govern intestinal calcium and phosphorus absorption, in bone to osteoclast differentiation and calcium reabsorption promoting mineralization of the bone matrix (St-Arnaud, 2008). It can either be produced in the skin by the photochemical conversion of the provitamins ergosterol and 7-dehydrocholesterol to cholecalciferol (D_3_) or absorbed from the diet in the intestinal tract. D_3_ is hydroxylated to 25-hydroxycholecalciferol (25-OH-D_3_) primarily in the liver and is circulated by the vitD binding protein (Haussler et al., 2013). This form is further hydroxylated in the kidneys, but also in the intestine, to the hormonally active form, 1α,25-dihydroxycholecalciferol (1,25-OH-D_3_) (Fleet and Schoch, 2010). The use of windowless houses in conventional broiler farms does not allow the skin to produce endogenous vitD, whilst raw materials in broiler diets contain little or no vitD (Atencio et al., 2005). As a result requirements are covered by means of dietary supplementation only (Waldenstedt, 2006). It has been demonstrated that dietary 25-OH-D_3_ is absorbed more efficiently than D_3_ in the upper portion of the intestine of broilers (Bar et al., 1980). At comparable levels of D_3_, the 25-OH-D_3_ isomer has been generally shown to improve performance and skeletal health (Cantor and Bacon, 1978; Fritts and Waldroup, 2003; Yarger et al., 1995b).

Historically, inclusion levels of vitD at a commercial level far exceed what is typically reported as the requirement by the NRC (200 IU/kg of feed) (Applegate and Angel, 2014). The latter, however, are based on old type, slower growing birds, whilst the current legal dietary limit in the European Union is 5,000 IU/kg feed. The basis for establishment of the upper limit is rather ambiguous and does not take into account bird genetic improvement; previous studies indicate adverse effects on performance, bone ash and renal calcification at levels of supplementation levels above 20,000 IU/kg (Baker et al., 1998; Browning and Cowieson, 2014; Yarger et al., 1995a). In commercial practice, dietary inclusion levels range between 2,000 and 5,000 IU/kg of feed (Whitehead et al., 2004; Leeson, 2007). In legislation there is no distinction about the form of vitD that can be added in the diet, whilst the European Food Safety Authority prohibits its addition to water (European Food Safety, 2009).

The objective of the present study was to re-evaluate the vitD requirements of 2 modern growing modern broiler genotypes offered Ca and P adequate diets. We hypothesized that at increasing levels, beyond the current legal limits, vitD supplementation of fast growing broilers will lead to improved skeletal integrity as measured by bone breaking strength (**BBS**), long bone mineralization, and better walking capacity. Furthermore, effects on bone mineralization were expected to be more pronounced at initial stages of growth when mineralization of the skeleton occurs most rapidly (Angel, 2007; Talaty et al., 2009). In addition, we hypothesized that if legal dietary maximum levels are to be maintained, partial substitution of D_3_ by 25-OH-D_3_ will be beneficial for bird skeletal integrity.

## MATERIAL AND METHODS

### Birds, Husbandry and Diets

All procedures were conducted under the UK Animals (Scientific Procedures) Act 1986 and were approved by the Animal Welfare and Ethical Review Body (AWERB) of Newcastle University. The trial was conducted in 2 rounds with an 8-wk interval. For each round, 144 male Ross 308 chicks and 144 male Ross 708 d old chicks were obtained from a commercial hatchery, vaccinated against avian infectious bronchitis virus (576 in total). Ross 308 is one of the most commonly used modern growing genotypes. Ross 708 is also a modern growing genotype, albeit displaying inferior FCR and ADG during the starter and grower period, commonly used in roaster market and is selected for high breast muscle yield. The same flocks of origin were used for both rounds, and were of 32 and 40 wk of age for the Ross 308 flock and of 36 and 44 wk of age for the Ross 708 flock, during the first and the second round respectively.

Birds were housed in a windowless thermostatically controlled building in 24 circular pens with a diameter of 1.2 m (1.13 m^2^), at an initial stocking density of 12 birds/pen. Pens were equipped with tube feeders and bell-drinkers and wood shavings were used as litter to a depth of 5 cm. Birds had *ad libitum* access to feed and water throughout the trial. Heat was supplemented with dull emitter ceramic bulbs. Temperature at pen level was monitored daily and maintained to meet Aviagen recommendations for spot brooding (Aviagen, 2014b), starting at 34°C at chick placement and gradually reduced to 21°C by 27 d, where it was maintained until 39 d of age when the trial was terminated. Light intensity at pen level was 80 lx whilst a lighting schedule of 23L:1D was applied for the first 7 d of age switched to 18L:6D for the remainder of the trial. All birds were individually wing-tagged at day 2 post-hatch.

A basal starter (day 1 to 10) grower (day 11 to 24) and finisher (day 25 to 39) diet were manufactured according to nutrition specifications (Aviagen, 2014a) apart from level and source of vitD (see below). The starter diet was offered in crumbled form and the grower and finisher diets in pelleted form (Table 1).

**Table 1.** Ingredient and analyzed chemical composition of the basal starter (day 1 to day 10 post-hatch), grower (day 11 to day 24 post-hatch), and finisher (day 25 to day 39 post-hatch) diets offered to the birds.

Item	Starter	Grower	Finisher
Ingredient (%)			
Wheat	47.8	51.5	54.8
Soybean meal (48% CP)	32.1	25.3	18.7
Corn	10	10	10
Full fat soybean	4.0	7.0	10
Dicalcium phosphate	1.90	1.66	1.46
Soy oil	1.84	2.32	2.86
Limestone	0.64	0.59	0.54
Vitamin and mineral premix^1^	0.4	0.4	0.4
DL-methionine	0.33	0.30	0.27
L-Lysine	0.27	0.25	0.23
Sodium bicarbonate (27%)	0.21	0.20	0.19
Sodium chloride (39%)	0.20	0.21	0.21
L-Threonine	0.15	0.12	0.10
Coccidiostat	0.07	0.07	0.06
Choline chloride (60%)	0.05	0.05	0.05
L-Valine	0.03	0.02	0.02
Xylanase^3^	0.02	0.02	0.01
Nutrient composition (%)^2^			
ME (kcal/kg) (calculated)	3,000	3,100	3,200
Crude protein	20.54	19.03	17.30
Ether extract	4.22	5.27	6.31
Calcium	0.93	0.84	0.83
Total phosphorus	0.74	0.70	0.66
Available phosphorus (calculated)	0.48	0.44	0.40
Sodium	0.16	0.16	0.17
Ash	5.23	4.78	4.37

^1,2^Provided per kilogram of feed vitamins, minerals, and digestible AA according to Aviagen Nutrient specifications (Aviagen, 2014a).

^3^Ronozyme WX, DSM Nutritional Products Ltd.

### Experimental Design

The experiment was 4 × 2 × 2 factorial design with dietary treatment, genotype, and round as the independent variables. Upon arrival, day old chicks of each of the Ross 308 and 708 genotypes were randomly allocated to 1 of 4 dietary treatments: a diet offering low level of D_3_ (LD; 1,000 IU/kg) which aimed at inducing a marginal vitD deficiency (Fritts et al., 2003), a diet offering medium level of D_3_, close to what is used in commercial practice (MD, 4,000 IU/kg), a diet offering high levels of D_3_, which is above the European Union legal limit of 5,000 IU/kg (HD; 7,000 IU/kg D_3_) and a diet offering the medium level of vitamin D_3_, where the majority of D_3_ was substituted by 25-OH-D_3_ (25MD; 1,000 D_3_+3,000 25-OH-D_3_ IU/kg). Each treatment (genotype × diet) had 3 replicate pens for each experimental round. Three birds per pen were selected based on their wing tag, 2 of which were sampled on day 10, day 24, and day 38 and 1 on day 11, day 25, and day 39. Pen body weight (**BW**) was measured at placement and pen FI and individual bird BW was measured at day 2, day 10, day 24, and day 38 of age and on day 11, day 25, and day 39 for birds dissected on those dates.

### Blood Sampling and Serum Levels of 25-OH-D3

The selected birds were individually weighed before blood-sampling via the wing vein and were subsequently euthanized with a lethal injection of sodium barbiturate (Euthatal, Merial Harlow, United Kingdom). Blood was placed in 5 mL serum tubes with serum clot activator, gel separator (BD Vacutainer, SST II Advance Plus Blood Collection Tubes—BD, Plymouth, United-Kingdom). Samples were allowed to clot for 1.5 h at room temperature and serum was collected, following centrifugation for 5 min at 1,300 × g in Eppendorf tubes and stored at −20°C pending analyses for serum levels of 25-OH-D_3_ using a commercially available ELISA kit specifically designed for chicken serum or plasma (MyBioSource, San Diego, CA).

### Bone Measurements

Following euthanasia the right tibia and femur were immediately dissected, defleshed, and stored in polystyrene air tight sealed bags at −20°C. Bones were thawed at 4°C in a walk-in fridge overnight and tibia and femur length and diameter at the center of the diaphysis were measured with digital callipers. Bones were subjected to a 3-point break test using an Instron testing machine (Instron 3340 Series, Single Column-Bluehill, Fareham Hants, UK). The testing support consisted of an adjustable 2-point block jig, spaced at 30 mm for tibias and 20 mm for femurs for the 10 and 11 d-old birds, and 30 mm for the older birds. The crosshead descended at 5 mm/min until a break was determined by measuring a reduction in force of at least 5%.

Following breaking strength, bones were split in 2 and the bone marrow was manually removed. Subsequently bones were soaked in petroleum ether for 48 h for lipid removal, were then placed in an oven at 105°C for 24 h and the dry bone weight was recorded. Samples were then ashed for 24 h at 600°C for the determination of ash weight (g) and ash percentage (%).

In birds dissected at day 11, 25, and 39 of age, tibia calcium and phosphorous ash content was measured by an inductively coupled plasma (ICP) emission spectroscopic method. Calcium and phosphorous standard for ICP (Fluka, Neu-Ulm, Switzerland) was prepared. Ashed sample of 0.5 g was weighed in a 100 mL beaker and 30 mL of 6 N HCl was added to each sample and placed in a fume cupboard to digest for 18 h at room temperature. Samples were then placed on a hot plate (100°C) in a fume cupboard and slowly brought to the boil and digested for 30 min with 50% (6 M) hydrochloric. The samples were cooled and quantitatively transferred to a 250 mL volumetric flask. Beakers were rinsed twice with 7 mL of 5% HCL to ensure complete transfer. The final volume was made up to 250 mL with 1% nitric acid solution. 1 mL of sample filtrate was pipetted into a 50 mL centrifuge tube and made up to 50 mL with 1% nitric acid solution. Samples were centrifuged at 1,500 rpm for 10 min at room temperature and introduced to an ICP spectrometer equipped with a CCD detector (ICP-OES; Varian Vista MPX, Varian, Palo Alto, CA, USA) and measured at wavelength 317.933 for calcium (Ca) and 213 nm for phosphorus (P) against prepared standards ranging from 1 to 100 ppm in calcium and phosphorus content.

### Gait Scoring

The remaining birds were individually assessed for their walking capacity at 37 d of age, using the 0 to 5 gait scoring system of Kestin et al. (1992), where a score of 0 represents a perfect gait and a score of 5 represents inability to stand. Briefly, gait score 0 (**GS** 0) describes a bird with no detectable gait abnormality; GS 1 birds have a slight walking defect; GS 2 birds have an identifiable defect; a GS 3 bird has an obvious gait abnormality; GS 4 birds have a severe gait defect, only walking when motivated. There were no birds with a GS of 5 in the current trial. The wing tag of each bird was noted and the bird was subsequently assessed by 3 independent observers, directly in their pens, after being herded away from the other birds. The score was then discussed between observers and the bird was possibly re-assessed until an agreement was reached.

### Statistics

Pen was the experimental unit for all data acquired at the end of starter, grower, and finisher period and all analysis were carried with SAS software (SAS 9.3, Cary, NC, USA). Femur and tibia ash % were averaged per pen for the 3 birds dissected at the end of each period. Tibia and femur dimensions (mm), and ash weight (g) obtained from the 3 sampled birds per pen at the end of each of the 3 periods were expressed as a proportion of individual BW at dissection (kg) and were then averaged per pen to account for differences in growth between genotypes, rounds, and sampling days. Expressing bone variables as a proportion of BW has been previously used in studies comparing genotypes differing in their growth potential (Shim et al., 2012). Analyzed Ca and P expressed as a percentage of tibia ash, Ca: P ratio and levels of 25-OH-D_3_ from 1 bird per pen dissected at day 11, day 25, and day 39 were analyzed with bird representing the pen. Data were statistically analyzed with the GLM procedure with dietary treatment, genotype, and round as the main effects including all 2-way and the 3-way interaction among and between the main effects. For the analysis of average pen BW and ADFI for the starter, grower and finisher stages the average pen BW obtained at day 2 of birds maintained until day 10, day 24, and day 38 post-hatch, respectively, was used as a covariate respectively to account for differences in starting BW among treatments and between rounds.

Initial analysis revealed the presence of round effects attributed to the older age of the maternal flocks at Round B, which resulted in a higher BW at placement in round B in comparison to round A (43.98 ± 0.24 vs. 38.06 ± 0.16 respectively). However, there were no significant interactions between round and the other factors for any of the measured variables. Therefore round and its 2-way and 3-way interaction with genotype and diet were excluded from the final model, which included only genotype, diet, and their interaction. When significant differences were detected, treatment means were separated and compared by the Tukey's multiple comparison test. For assessing the normality of the residuals the Shapiro–Wilk test was used. Significance was determined at *P <* 0.05. Values are expressed as model predicted means along with their pooled SEM.

Additional polynomial contrasts were performed to study the linear and quadratic responses of bone variables and serum 25-OH-D_3_ levels to increased levels of D_3_ (1,000, 4,000, and 7,000 IU/kg). Single degree of freedom contrasts were carried to compare the 25MD treatment with the MD and HD treatment and the MD with the HD treatments, on bone variables and serum 25-OH-D_3_ levels.

## RESULTS

### Performance and GS

Both diet and its interaction with genotype did not affect any of the performance variables. Main effects of genotype and diet are presented in Table 2. Genotype significantly affected BW (*P <* 0.05) with birds of the Ross 708 genotype achieving lower BW than the Ross 308 at the end of the starter (*P <* 0.05), grower (*P <* 0.01), and finisher period (*P <* 0.0001). At the same time, they had significantly higher FCR during the grower period (*P <* 0.05).

**Table 2.** Main effect of genotype (Ross 308 or 708) and dietary treatments on body weight (BW), average daily feed intake (ADFI), and feed conversion ratio (FCR) over the starter (day 1 to 10), grower (day 11 to 24), and finisher period (day 25 to 38) and on gait score (GS) at day 37 of age. LS means of BW and ADFI are adjusted for BW at day 2 post-hatch (cvBW) which was used as a covariate.

	BW (g)			ADFI (g)			FCR			GS
	Day 10	Day 24	Day 38	Day 1 to 10	Day 11 to 24	Day 25 to 38	Day 1 to 10	Day 11 to 24	Day 25 to 38	Day 37
Genotype										
Ross 308	334.8	1516.6	3349.8	36.2	109.9	200.0	1.23	1.30	1.53	1.97
Ross 708	329.2	1472.9	3201.7	36.2	108.8	192.8	1.25	1.33	1.56	2.24
SEM	1.5	9.9	24.4	0.3	1.1	2.6	0.01	0.01	0.02	0.09
Diet										
LD	332.2	1518.2	3293.2	36.9	111.9	195.8	1.27	1.32	1.55	2.36^a^
MD	329.0	1475.8	3276.6	35.8	107.1	197.6	1.24	1.31	1.54	2.23^a,b^
HD	333.4	1501.5	3305.8	36.0	110.2	202.5	1.23	1.32	1.57	2.09^a,b^
25MD	333.5	1483.6	3227.5	35.9	108.3	189.8	1.23	1.32	1.52	1.72^b^
SEM	2.2	14.0	34.5	0.5	1.5	3.7	0.01	0.01	0.02	0.13
Source	*Probabilities*									
Genotype	**<0.01**	**<0.01**	**<0.001**	0.988	0.542	0.101	0.078	**<0.05**	0.177	**<0.05**
Diet	0.120	0.763	0.607	0.390	0.293	0.113	0.101	0.911	0.510	**<0.01**
cvBW	**<0.001**	**<0.05**	0.082	0.848	0.990	0.165	*NA*	*NA*	*NA*	*NA*

^a,b^Means in each column with different superscripts are significantly different (*P* < 0.05).

Abbreviations: LD (low level of D_3_; 1,000 IU/kg), MD (medium level of D_3_; 4,000 IU/kg), HD (high level of D_3_; 7,000 IU/kg D^3^), 25MD (medium level consisting of 1,000 D_3_+3,000 25-OH- D_3_ IU/kg).

Genotype and diet did not interact for GS. Birds of the Ross 708 genotype had significantly higher GS (*P <* 0.05) than birds of the Ross 308 genotype. Diet significantly affected GS (*P <* 0.01), with birds on the LD treatment achieving significant higher GS than birds on the 25MD treatment.

### Bone Measurements

There was no significant interaction between genotype and dietary treatment on any of the bone variables tested. Main effects of genotype and diet of the GLM model, as well as linear and quadratic effects and contrasts on femur and tibia variables are presented in Tables 3 and 4, respectively.

**Table 3.** Main effect of genotype (Ross 308 or 708) and dietary treatments (GLM), linear, quadratic and single degree of freedom contrasts on femur dimensions, bone breaking strength (BBS), and ash weight (g) expressed as a proportion of BW (kg) at dissection and ash percentage (%).

Femur	Length (mm/kg BW)			Width (mm/kg BW)			Ash (g/kg BW)			BBS (N/kg BW)			Ash (%)		
Day post-hatch	Day 10,11	Day 24,25	Day 38,39	Day 10,11	Day 24,25	Day 38,39	Day 10,11	Day 24,25	Day 38,39	Day 10,11	Day 24,25	Day 38,39	Day 10,11	Day 24,25	Day 38,39
Genotype															
Ross 308	117.8	41.9	24.7	11.35	5.06	3.13	0.698	0.782	0.748	274.6	159.4	102.0	43.4	45.5	45.2
Ross 708	119.6	42.5	25.6	11.71	5.10	3.27	0.699	0.764	0.732	283.5	165.0	105.0	43.6	45.3	45.6
SEM	1.2	0.4	0.2	0.12	0.05	0.03	0.007	0.007	0.007	4.1	3.3	1.9	0.2	0.2	0.2
Diet															
LD	117.3	41.6	24.6	11.44	5.02	3.17	0.680	0.749^a^	0.738^a,b^	274.7	156.7	98.1^a^	42.8^a^	44.6^a^	45.1
MD	120.4	42.8	25.2	11.47	5.10	3.20	0.707	0.775^a,b^	0.719^a^	269.1	164.2	101.3^a^	43.5^a,b^	45.2^a,b^	45.3
HD	120.3	41.9	25.3	11.68	5.05	3.22	0.701	0.776^a,b^	0.744^a,b^	281.3	160.5	103.9^a,b^	43.5^a,b^	46.0^b^	45.3
25MD	116.9	42.5	25.5	11.52	5.17	3.20	0.707	0.793^b^	0.759^b^	291.0	167.5	110.9^b^	44.2^b^	45.8^b^	45.8
SEM	1.7	0.5	0.3	0.18	0.07	0.04	0.010	0.010	0.010	5.8	4.7	2.7	0.3	0.3	0.3
Source	*Probabilities*														
Genotype	0.314	0.290	**<0.01**	**<0.05**	0.550	**<0.01**	0.932	0.089	0.109	0.155	0.257	0.269	0.372	0.436	0.092
Diet	0.301	0.344	0.099	0.773	0.388	0.799	0.308	**<0.05**	0.056	0.083	0.441	**<0.05**	**<0.05**	**<0.01**	0.336
Contrasts															
D3 level															
Linear	0.167	0.754	0.096	0.345	0.725	0.324	0.240	0.063	0.668	0.219	0.607	0.076	0.444	**<0.01**	0.588
Quadratic	0.401	0.149	0.414	0.679	0.394	0.867	0.225	0.298	**<0.05**	0.218	0.384	0.903	0.237	0.800	0.882
MD vs. 25MD	0.154	0.718	0.412	0.834	0.468	0.939	0.922	0.281	**<0.05**	**<0.05**	0.643	**<0.05**	0.224	0.159	0.206
MD vs. HD	0.979	0.199	0.882	0.377	0.541	0.713	0.639	0.965	0.096	0.148	0.608	0.520	0.613	0.091	0.890
HD vs. 25MD	0.161	0.350	0.500	0.498	0.186	0.657	0.710	0.301	0.304	0.243	0.332	0.089	0.090	0.764	0.258

^a,b^Means in each column with different superscripts are significantly different (*P* < 0.05).

Abbreviations: LD (low level of D_3_; 1,000 IU/kg), MD (medium level of D_3_; 4,000 IU/kg), HD (high level of D_3_; 7,000 IU/kg D^3^), 25MD (medium level consisting of 1,000 D_3_+3,000 25-OH- D_3_ IU/kg).

**Table 4.** Main effect of genotype (Ross 308 or 708) and dietary treatments (GLM), linear, quadratic and single degree of freedom contrasts on tibia dimensions, bone breaking strength (BBS), and ash weight (g) expressed as a proportion of BW (kg) at dissection and ash percentage (%).

Tibia	Length (mm/kg BW)			Width (mm/kg BW)			Ash (g/kg BW)			BBS (N/kg BW)			Ash (%)		
Day post-hatch	Day 10,11	Day 24,25	Day 38,39	Day 10,11	Day 24,25	Day 38,39	Day 10,11	Day 24,25	Day 38,39	Day 10,11	Day 24,25	Day 38,39	Day 10,11	Day 24,25	Day 38,39
Genotype															
Ross 308	156.1	56.7	34.4	10.49	4.55	2.85	1.001	1.125	1.186	17.4	198.1	126.2	43.6	46.6	46.3
Ross 708	159.1	58.0	35.9	10.44	4.51	2.85	0.992	1.087	1.140	17.3	192.3	127.1	44.0	46.4	46.9
SEM	1.6	0.5	0.3	0.11	0.05	0.03	0.008	0.008	0.013	0.3	3.9	3.1	0.3	0.2	0.2
Diet															
LD	155.8	56.4	34.7	10.43	4.36^a^	2.81	0.989	1.077^a^	1.161	17.3	186.1^a^	122.5	43.0	46.0	46.2
MD	157.5	58.2	35.2	10.36	4.65^b^	2.83	0.999	1.104^a,b^	1.134	16.6	192.2^a,b^	124.6	44.4	46.6	46.5
HD	160.9	57.0	35.1	10.69	4.43^a,b^	2.83	0.997	1.112^a,b^	1.154	17.3	193.8^a,b^	126.2	44.0	46.7	46.5
25MD	156.2	57.9	35.7	10.37	4.68^b^	2.92	1.003	1.129^a^	1.203	18.2	208.7^b^	133.3	43.8	46.9	47.2
SEM	2.3	0.7	0.4	0.15	0.07	0.05	0.011	0.012	0.019	0.4	5.5	4.3	0.4	0.2	0.3
Source	*Probabilities*														
Genotype	0.194	0.093	**<0.01**	0.758	0.629	0.956	0.418	**<0.01**	**<0.05**	0.936	0.292	0.826	0.378	0.384	0.055
Diet	0.388	0.292	0.374	0.394	**<0.01**	0.335	0.848	**<0.05**	0.089	0.060	**<0.05**	0.335	0.094	0.054	0.234
Contrasts															
D3 level															
Linear	0.129	0.577	0.464	0.228	0.425	0.780	0.565	**0.050**	0.798	0.866	0.324	0.577	0.065	0.052	0.625
Quadratic	0.764	0.142	0.577	0.284	**<0.01**	0.906	0.586	0.523	0.306	0.173	0.737	0.975	0.077	0.488	0.819
MD vs. 25MD	0.707	0.770	0.370	0.979	0.732	0.118	0.823	**<0.05**	**<0.05**	**<0.05**	0.061	0.151	0.345	0.318	0.123
MD vs. HD	0.309	0.242	0.899	0.158	**<0.05**	0.970	0.889	0.255	0.433	0.255	0.852	0.783	0.548	0.664	0.963
HD vs. 25MD	0.168	0.375	0.308	0.165	**<0.05**	0.126	0.717	0.190	0.062	0.190	0.089	0.241	0.728	0.568	0.134

^a,b^Means in each column with different superscripts are significantly different (*P* < 0.05).

Abbreviations: LD (low level of D_3_; 1,000 IU/kg), MD (medium level of D_3_; 4,000 IU/kg), HD (high level of D_3_; 7,000 IU/kg D^3^), 25MD (medium level consisting of 1,000 D_3_+3,000 25-OH- D_3_ IU/kg).

#### Genotype.

Birds of the Ross 708 growing genotype had significantly longer femurs per unit of BW (*P <* 0.01) at the end of the finisher period, which were significantly wider at the end of the starter (*P <* 0.05) and finisher periods (*P <* 0.01) in comparison to those of the Ross 308 genotype. Tibias were significantly longer (*P <* 0.001) per unit BW in birds of the Ross 708 genotype at the end of the finisher period than in Ross 308 birds. Tibia of birds of the Ross 708 line yielded less ash weight at the end of the grower (*P <* 0.01) and finisher periods (*P <* 0.01).

#### Diet.

Femur ash weight was affected by diet (*P <* 0.05) at the end of the grower period, being significantly higher (*P <* 0.05) for birds on the 25MD diet in comparison to the LD treatment. Femur BBS was significantly affected during the finisher period (*P <* 0.01), being significantly higher for the 25MD in comparison to LD birds (*P <* 0.05). Femur ash % was significantly affected by diet at the end of the starter and grower periods; it was significantly higher (*P <* 0.05) for birds on the 25MD than on the LD treatment (*P <* 0.05) and for birds in both HD and 25MD treatments in comparison to birds on the LD treatment (*P <* 0.05).

Tibia width was significantly affected by diet (*P <* 0.01), being significantly higher for both 25MD and MD birds (*P <* 0.05) than LD birds at the end of the grower period. Tibia ash weight was significantly affected by diet (*P <* 0.05), being higher in 25MD birds in comparison to LD birds at the end of the grower period (*P <* 0.05) and tended to be affected at the end of the finisher period being numerically higher for birds on the 25MD treatment. Tibia BBS was significantly affected (*P <* 0.05) at the end of the grower period being higher for 25MD birds than LD birds (*P <* 0.05).

#### Linear and Quadratic Effects and Contrasts.

Significant linear effects (*P <* 0.05) were obtained at the end of the grower period for femur ash % and for tibia ash weight, both increasing at increasing levels of D_3_. A quadratic effect was obtained at the end of the grower period for tibia width (*P <* 0.01) and at the end of the finisher period for femur ash weight (*P <* 0.05). Contrasts between MD and 25MD treatments revealed that 25MD treatment resulted in significantly higher femur ash weight at the end of the finisher period and tibia ash weight at the end of the grower and finisher periods weight (*P <* 0.05). Similarly, higher tibia BBS was achieved at the end of the grower period for 25MD in comparison to the MD treatment weight (*P <* 0.05). There was a significant linear effect of diet on tibia Ca percentage on day 11, with its levels increasing as vitD increased. However, no significant differences were revealed when contrasting 25MD with HD or MD with HD other than a significantly lower tibia width for HD in comparison to both other treatments (*P <* 0.05).

### Serum 25-OH-D3

There was no significant effect of genotype and no significant interaction between genotype and dietary treatment on serum 25-OH-D_3_ levels of birds dissected at day 11, day 25, and day 39 of age. Main effects of genotype and diet of the GLM model, as well as linear and quadratic effects and contrasts, are presented in Table 5. Dietary treatment significantly affected (*P <* 0.001) serum 25-OH-D_3_ levels at both day 11 and day 25 of age (*P <* 0.0001) (Table 5); the effect of dietary levels of D3 was linear, with increasing level of D3 leading to increased serum 25-OH-D_3_ levels. 25MD birds contained higher levels of serum 25-OH-D_3_ than MD birds on d11 and d25.

**Table 5.** Main effect of genotype (Ross 308 or 708) and dietary treatments (GLM), linear, quadratic and single degree of freedom contrasts on tibia calcium (Ca) and phosphorus (P) expressed as a proportion of bone ash, their ratio and serum levels of 25-OH-D3 at day 11, day 25, and day 39 post-hatch.

Variable	Ca (% Ash)			P (% Ash)			Ca:P ratio			25-OH-D3 (ng/mL)		
Day post-hatch	Day 11	Day 25	Day 39	Day 11	Day 25	Day 39	Day 11	Day 25	Day 39	Day 11	Day 25	Day 39
Genotype												
Ross 308	34.48	34.86	35.88	17.57	17.43	17.75	1.96	2.00	2.02	60.83	74.22	72.23
Ross 708	34.36	34.67	35.73	17.56	17.28	17.71	1.95	2.01	2.02	63.46	70.61	74.65
SEM	0.14	0.14	0.14	0.08	0.06	0.062	0.01	0.01	0.01	2.75	2.78	2.11
Diet												
LD	33.99	34.83	35.89	17.52	17.43	17.86	1.94	2.00	2.01	47.60^a^	63.18^a^	69.80
MD	34.48	34.63	35.72	17.55	17.27	17.66	1.96	2.01	2.02	60.34^a,b^	72.34^a,b^	71.87
HD	34.51	34.80	35.55	17.57	17.35	17.65	1.96	2.01	2.01	67.48^b^	75.85^b^	74.92
25MD	34.71	34.80	36.08	17.63	17.37	17.75	1.96	2.00	2.03	73.17^b^	78.30^b^	77.19
SEM	0.20	0.19	0.20	0.11	0.09	0.09	0.01	0.01	0.01	3.90	3.94	2.99
Source	*Probabilities*											
Genotype	0.518	0.316	0.445	0.933	0.087	0.760	0.430	0.367	0.684	0.494	0.345	0.436
Diet	0.122	0.892	0.277	0.929	0.701	0.325	0.345	0.927	0.336	**<0.001**	**<0.05**	0.349
Contrasts												
D3 level												
Linear	**<0.05**	0.910	0.252	0.769	0.574	0.132	0.262	0.549	0.726	**<0.001**	**<0.05**	0.241
Quadratic	0.278	0.464	0.996	0.979	0.327	0.455	0.385	0.781	0.345	0.494	0.552	0.897
MD vs. 25MD	0.461	0.610	0.217	0.636	0.523	0.613	0.805	0.915	0.915	**<0.05**	0.290	0.228
MD vs. HD	0.930	0.559	0.555	0.901	0.548	0.898	0.833	0.950	0.950	0.247	0.531	0.486
HD vs. 25MD	0.515	0.952	0.073	0.727	0.957	0.529	0.640	0.867	0.867	0.354	0.662	0.602

^a,b^Means in each column with different superscripts are significantly different (*P* < 0.05).

Abbreviations: LD (low level of D_3_; 1,000 IU/kg), MD (medium level of D_3_; 4,000 IU/kg), HD (high level of D_3_; 7,000 IU/kg D^3^), 25MD (medium level consisting of 1,000 D_3_+3,000 25-OH- D_3_ IU/kg).

## DISCUSSION

In the present study we assessed the effects of vitD supplementation by offering a diet with suboptimal levels of D_3_ (LD), diets with levels close to commercial recommendations where the additional vitD was offered either as D_3_ (MD) or as 25-OH-D_3_ (25MD), and a diet above commercially recommended levels, and European Union allowed levels offered as D_3_ (HD). We hypothesized that a linear increase of D_3_ supplementation levels will result in higher skeletal integrity, as measured by BBS, bone mineralization and better walking capacity, which will be linked with higher levels of serum 25-OH-D_3_. In addition, we expected that partial substitution of D_3_ by 25-OH-D_3_ treatment at commercial supplementation levels would affect measured variables to a higher degree as it has been shown to be metabolically more potent on a per unit basis than D_3_ (Fritts et al., 2003). Linear effects of vitD supply in the form of D_3_ were observed on the level of femur ash (%) and tibia ash (g) at the end of the growing period. However, contrary to our hypothesis, vitD supplementation levels above 5,000 IU/kg (HD) in the form of D_3_ did not confer substantial benefits on tibia and femur mineralization in comparison to commercially used levels (MD). Partial substitution of D_3_ by 25-OH-D_3_ at commercial levels of supplementation leads to improved aspects of long bone mineralization. Nonetheless, the majority of dietary effects derived from the differences observed between 25MD and LD treatment groups, the latter leading to a state of marginal deficiency in regards to bone mineralization according to hypothesis.

In the present study, performance was largely unaffected by level or source of vitD supplementation. Although, positive effects of vitD supplementation above NRC recommendations are common (Baker et al., 1998; Fritts and Waldroup, 2003; Whitehead et al., 2004; Rao et al., 2006), the range at which effects are observed and the nature of the parameters affected vary substantially amongst studies. Waldroup et al. (1965) were the first to illustrate that vitD requirements vary according to Ca and P dietary contents and their ratio. The absence of a significant effect on performance in our experiment, is possibly related to the adequate levels of Ca and P which were supplemented in the diets, as efficacy of vitD has been shown to be intrinsically related to their nutrient availability (Rennie et al., 1995; Ledwaba and Roberson, 2003; Whitehead et al., 2004; Rao et al., 2006, 2007, 2008). One would expect improved performance in the 25MD treatment during the starter period in comparison to the D_3_ treatments owing to the relatively fat independent absorption of 25-OH-D3 as opposed to D_3_ (Sitrin and Bengoa, 1987; Borel et al., 2015), and the limited ability of young chicks to digest and absorb fat (Tancharoenrat et al., 2013), but this was not supported by our findings.

In the absence of effects of our dietary treatments on performance, femur, and to a lesser extent tibia, showed generally increased mineral deposition (ash, g) and increased mineralization (ash, %). These effects were observed at the end of the starter and grower period for femur, whilst effects only approached a tendency for tibia. This is not surprising as increased mineralization in the absence of effects on performance is commonly observed in response to supplementation with Ca, and to a lesser extend P, as well as with vitD (Bar et al., 2003; Angel et al., 2006). The majority of absorbed Ca and P is primarily required for bone formation (Adedokun and Adeola, 2013). An adequate level of dietary Ca is essential for the bone deposition and mineralization of P (Rousseau et al., 2012), whilst rapid bone growth, requires adequate Ca and P supply (Williams et al., 2000b). VitD regulates calcium and phosphorus metabolism mainly by enhancing intestinal calcium and phosphate absorption and renal reabsorption whilst it also stimulates osteoclast differentiation and calcium reabsorption from bone and promotes mineralization of the bone matrix (St-Arnaud, 2008; Bikle, 2012; Haussler et al., 2013). It has been estimated that during the initial stages of growth the load applied to the skeleton due to a quadratic increase in BW gain may be by as much as 32-fold imposing a requirement for rapid skeletal adaptation (Yair et al., 2012). The rate of growth and mineralization of the skeleton occurs most rapidly during the first 2 wk of age in the growing chick (Angel, 2007) and continues until 4 wk of age, whereas after this period tibias grow in length, width, and surface but this is not accompanied by increases in bone mineral density and bone mineral content (Talaty et al., 2009).

The stronger responses to dietary supplementation with 25-OH-D_3_ and to increasing dietary levels of D_3_ at the end of the starter and grower periods on femur ash % are in accordance to the higher earlier mineralization rate of the femur as compared to tibia (Applegate and Lilburn, 2002) and are in agreement with the suggestion that it is a better marker to assess responses of dietary treatments on bone mineralization (Angel et al., 2006). Dietary effects on other measurements of skeletal integrity were less consistent; effects on BBS were significant in the grower period for the tibia but not on the femur. Surprisingly, there was an effect of diet on femur BBS at the end of the finisher period with 25MD diets having significantly higher BBS than both LD and MD dietary treatments. This is contrary to our expectation as efficacy of the metabolites should be reduced by the end of the finisher period as it has been previously demonstrated that vitD supplementation is more critical during the initial stages of growth for bone development (Whitehead et al., 2004). On the other hand, serum levels of 25-OH-D_3_ were responsive to the dietary treatments at the end of the starter and grower stage, but absent by the end of the finisher stage. One could interpret that there was a state of adequacy at the later stages of growth, despite the observed skeletal effects later in the finisher period (Whitehead et al., 2004). However, it is contrary to conventional expectation as circulating levels of 25-OH-D_3_ should increase more rapidly in response to 25-OH-D_3_ supplementation over time (Yarger et al., 1995b). In the starter diets both 25MD and HD treatments had significantly higher values of 25-OH-D_3_ than LD, suggesting that at least for the starter phase one could increase D_3_ supplementation level above EU recommendations in order to improve vitD adequacy. Therefore, dietary treatment effects on ash weight as a proportion of BW at the end of the finisher period are more likely a consequence of higher levels of supplementation earlier in the starter and grower periods.

In the present study vitD supplementation was associated with a lower GS in LD birds, in agreement with Sun et al., (2013). Contrary to these findings, Venalainen et al. (2006) did not find any effect of increasing dietary Ca and P content on the walking ability of broilers, although increased Ca and P supplementation affected tibia mineral deposition (Venalainen et al., 2006). Even if the incidence of tibial dyschondroplasia has been reduced in modern broiler populations (Kapell et al., 2012), it is possible that development of tibial dyschondroplasia may have been augmented by our LD treatments as vitamin D supplementation has been shown to reduce its prevalence (Elliot et al., 1995; Berry et al., 1996; Rennie et al., 1995; Zhang et al., 1997; Ledwaba et al., 2003). Furthermore, one cannot discard the protective role that 25-OH-D_3_ has been shown to exert in the development of bacterial chondronecrosis, which is considered the most prevalent cause of lameness, in comparison to D_3_ (Wideman et al., 2015). Therefore, a deterioration of GS in the LD treatment may be linked to impaired bone long bone development as indicated by the markers of mineralization, but also to the incidence and severity of skeletal disorders although these were not measured in the present study.

Higher growth rates have been associated with altered Ca: P ratios in cortical bone (Williams et al., 2000a) and at the same time altered Ca: P ratio has been found in broiler bones suffering from leg pathologies (Thorp and Waddington, 1997) although their concentration as components of hydroxyapatite is considered to be stable at a molar ration of 2:1 (Field, 2000). It has been suggested that although bone ash % may be decreased by low Ca or P diets, the Ca concentration in ash still remains constant close to 37% whilst deviations from this rule reflect differences in bone preparation and analysis (Field, 2000). However, in the present study a reduced Ca percentage was observed at the end of the starter period in LD birds, which nonetheless was not associated with penalties on other markers of mineralization. More insight into the effects of vitD on Ca: P ratio in reference to bone development may have been gained had we focused on cortical bone samples rather than the ash of both trabecular and cortical bone.

We hypothesized that reduced growth rates and feed efficiency early in the starter period and grower period would lead to reduced requirements of D_3_. When assessing effects of genotype on growth rate, it should be noted that birds performed significantly higher than reported in the performance objectives (3223 and 3434 vs. 2472 and 2599, respectively at day 38 of age; Aviagen 2014c,d). In the present study birds of the Ross 308 showed increased BW at the end of the starter, grower, and finisher periods according to expectations. This was not accompanied by increases in ADFI. According to the performance objectives Ross 708 should have displayed a higher initial FCR whilst differences between genotypes should be smaller at the end of the grower and finisher periods. In our study there was a tendency for a lower FCR of Ross 308 at the starter period whilst this difference was significant at the end of the grower period. In terms of their skeletal traits Ross 708 displayed longer bones at the end of the finisher period whilst femurs were wider. However, tibias carried less ash weight as a proportion of BW at the end of the grower and finisher periods. On the other hand, there was a strong tendency (*P =* 0.055) for tibia ash % to be higher at the end of the finisher period for Ross 708 indicating that at older ages bones of this genotype may be more mineralized. This genotype is maintained until a later age and a higher degree of mineralization may have resulted in better skeletal integrity at later stages of growth. Despite observed differences in ash values, BBS, which is indicative of the load bearing capacity of the long bones (Rath et al., 2000) was ultimately similar for both strains. The absence of genotype and diet interactions indicates that subtle differences in growth rate and FCR did not influence the requirements of the 2 genotypes in vitD. Previous research comparing the 2 genotypes has similarly shown that their requirements for P are not significantly different (Persia and Saylor., 2006). Although the GS of Ross 708 was inferior to that of Ross 308 broilers, the difference between the 2 genotypes was relatively small (1.97 vs. 2.23) and likely reflects different selection objectives of the 2 genotypes as Ross 708 is more heavily selected for breast muscle. According to performance objectives at the slaughter weight achieved at the end of this study, Ross 708 has 1.7% of additional breast muscle in comparison to the Ross 308 genotype whilst the weight of thigh is similar and that of drumstick is reduced as a proportion of the eviscerated carcass. It has been suggested that the rapid growth of breast muscle moves the center of gravity forward (Corr et al., 2003b). In a field evaluation trial, birds of the same commercial line with a higher GS, displayed differences in skeletal conformity traits related to breast development such as different breast angle (Skinner-Noble and Teeter, 2009). Finally, studies assessing the effects of selection on locomotion have clearly illustrated that differences in breast muscle conformity traits have pronounced effects on locomotion patterns and gait dynamics (Paxton et al., 2013, 2014). It is noteworthy that GS is a subjective method of evaluation of the bird's walking capacity and gait patterns of the 2 genotypes were substantially different; the Ross 708 has a wider stride which is in line with the prevalence of more breast muscle. As a result it walks *differently* and tends to get a higher GS (Sakkas, personal observation).

In conclusion, although dietary treatment effects on performance were absent, offering a diet which included 25-OH-D_3_ led to consistent improvements in bone mineralization. These were seen as improvements in femur and tibia ash content (per unit of BW) and BBS. The effects were more obvious at the end of the grower period (∼25 d of age) rather than at the end of starter and finisher periods. The effects of the high vitamin D_3_ on bone mineralization were less consistent and not statistically different from either the effects of MD or 25MD. It is possible that this was due to the statistical power of our experiment. Our results do not suggest that current maximum legal limits of dietary vitD inclusion need to be re-evaluated.

## Notes

Author disclosures: The authors have no conflicts of interest.

Supported by ProHealth. The PROHEALTH project has received funding from the European Union's Seventh Framework Programme for research, technological development, and demonstration under grant agreement no. 613574.
